# Chinese expert consensus on the surgical treatment of femoral neck fracture by direct anterior approach hip arthroplasty for elderly patients (2025 edition)

**DOI:** 10.1186/s42836-026-00368-9

**Published:** 2026-02-13

**Authors:** Xiaojun Man, Zhaxi Mima, Zhonghua Xu, Zaiyang Liu, Jie Li, Jun Zhang, Xia Zhang, Min Wang, Fei Luo, Guangxing Chen, Yan Xiong, Guodong Liu, Dawei Zhang, Xiaorui Cao, Guoqiang Zhang, Wei Chai, Pingyue Li, Nirong Bao, Xiaoqing He, Shenghu Zhou, Bo Wu, Wenwei Qian, Weiguo Wang, Yixin Zhou, Hao Tang, Hu Li, Chuan He, Yunsu Chen, Huiwu Li, Wei Huang, Ning Hu, Mao Nie, Feng Xie, Zhidong Cao, Zongke Zhou, Ye Ye, Xianzhe Liu, Weihua Xu, Xinzhan Mao, Jie Xie, Li Cao, Xiaogang Zhang, Boyong Xu, Pei Yang, Wei Wang, Xiaofeng Li, Eryou Feng, Zhen Zhang, Baoyi Liu, Hui Li, Yuanchen Ma, Li Sun, Zhifeng Zhang, Shuo Geng, Luobu Zhaxi, Jinmei Awang, Li Xiong, Kunzheng Wang, Chen Zhu, Yuan Zhang

**Affiliations:** 1 Department of Orthopedics, Xinqiao Hospital, Army Medical University, Chongqing, 400037 China; 2 Shigatse Branch Hospital of Xinqiao Hospital, Army Medical University, Shigatse, 857099 Tibet China; 3 Department of Orthopedics, Southwest Hospital, Army Medical University, Chongqing, 400038 China; 4 Department of Orthopedics, Daping Hospital, Army Medical University, Chongqing, 400042 China; 5 Department of Orthopedics, Xijing Hospital, Air Force Medical University, Xi’an, 710032 Shaanxi China; 6 Department of Orthopedics, Chinese PLA General Hospital, Beijing, 100853 China; 7 Department of Orthopedics, General Hospital of Southern Theater Command, PLA, Guangzhou, 510010 Guangdong China; 8 Department of Orthopedics, General Hospital of Eastern Theater Command, PLA, Nanjing, 210016 Jiangsu China; 9 Department of Orthopedics, The 920th Hospital of Joint Logistics Support Force of PLA, Kunming, Yunnan 650032 China; 10 Department of Orthopedics, The 940th Hospital of Joint Logistics Support Force of PLA, Lanzhou, Gansu 730050 China; 11 Department of Orthopedics, The 960th Hospital of Joint Logistics Support Force of PLA, Jinan, Shandong 250031 China; 12 Department of Orthopedics, Peking Union Medical College Hospital, Beijing, 100730 China; 13 Department of Orthopedics, China-Japan Friendship Hospital, Beijing, 100029 China; 14 Department of Orthopedics, Beijing Jishuitan Hospital, Beijing, 100035 China; 15 Department of Orthopedics, Peking University People’s Hospital, Beijing, 100044 China; 16 Department of Orthopedics, Ruijin Hospital Affiliated to Shanghai Jiao Tong University School of Medicine, Shanghai, 200025 China; 17 Department of Orthopedics, Shanghai Sixth People’s Hospital Affiliated to Shanghai Jiao Tong University School of Medicine, Shanghai, 200233 China; 18 Department of Orthopedics, Shanghai Ninth People’s Hospital Affiliated to Shanghai Jiao Tong University School of Medicine, Shanghai, 200011 China; 19 Department of Orthopedics, The First Affiliated Hospital of Chongqing Medical University, Chongqing, 400042 China; 20 Department of Orthopedics, The Second Affiliated Hospital of Chongqing Medical University, Chongqing, 400010 China; 21 Department of Orthopedics, The Third Affiliated Hospital of Chongqing Medical University, Chongqing, 401120 China; 22 Department of Orthopedics, Chongqing University Central Hospital (Chongqing Emergency Medical Center), Chongqing, 400014 China; 23 Department of Orthopedics, West China Hospital, Sichuan University, Chengdu, 610041 Sichuan China; 24 Department of Orthopedics, Sichuan Orthopedic Hospital, Chengdu, 610041 Sichuan China; 25 Department of Orthopedics, Union Hospital, Tongji Medical College, Huazhong University of Science and Technology, Wuhan 430022, Hubei, China; 26 Department of Orthopedics, The Second Xiangya Hospital of Central South University, Changsha, 410012 Hunan China; 27 Department of Orthopedics, The First Affiliated Hospital, Zhejiang University School of Medicine, Hangzhou, 310003 Zhejiang China; 28 Department of Orthopedics, The First Affiliated Hospital of Xinjiang Medical University, Urumqi, 830054 Xinjiang China; 29 Department of Orthopedics, The Second Affiliated Hospital of Xi’an Jiaotong University, Xi’an, 710004 Shaanxi China; 30 ⁰Department of Orthopedics, The First Affiliated Hospital of Nanchang University, Nanchang, 330006 Jiangxi China; 31 Department of Orthopedics, Fujian Medical University Union Hospital, Fuzhou, 350001 Fujian China; 32 Department of Orthopedics, Affiliated Hospital of Dalian Medical University, Dalian, 116011 Liaoning China; 33 Department of Orthopedics, Zhongshan Hospital Affiliated to Dalian University, Dalian, 116001 Liaoning China; 34 Department of Orthopedics, Honghui Hospital Affiliated to Xi’an Jiaotong University, Xi’an, 710054 Shaanxi China; 35 Department of Orthopedics, Guangdong Provincial People’s Hospital, Guangzhou, 519041 Guangdong China; 36 Department of Orthopedics, Guizhou Provincial People’s Hospital, Guiyang, 550499 Guizhou China; 37 Department of Orthopedics, The Second Affiliated Hospital of Inner Mongolia Medical University, Hohhot, 010110 Inner Mongolia China; 38 Department of Orthopedics, The Second Affiliated Hospital of Harbin Medical University, Harbin, 150081 Heilongjiang China; 39 Department of Orthopedics, People’s Hospital of Lhasa City, Tibet Autonomous Region, Lhasa, 850000 Tibet China; 40 Department of Orthopedics, General Hospital of Tibet Military Command, PLA, Lhasa, 850000 Tibet China; 41 Department of Orthopedics, The First Affiliated Hospital of University of Science and Technology of China, Hefei, 230001 Anhui China; 42 Chongqing Municipal High-End Medical Talent Studio, Chongqing, 400037 China

**Keywords:** Geriatric, Femoral neck fracture, Hip arthroplasty, Direct anterior approach, Robot

## Abstract

Geriatric femoral neck fracture (FNF) represents a pressing global health challenge, imposing substantial burdens on medical resources while being associated with high complication rates and suboptimal clinical outcomes. In recent years, the direct anterior approach (DAA) has emerged as a mainstream surgical strategy for hip joint arthroplasty (HJA) in developed countries, owing to its minimally invasive nature, neuromuscular-sparing anatomical interval, preservation of soft tissue integrity, and alignment with enhanced recovery after surgery principles, collectively contributing to significant improvements in clinical outcomes of HJA for elderly patients with FNF. However, no unified consensus exists regarding standardized techniques and procedural protocols for DAA-HJA in this specific patient population. Consequently, there is an urgent need to develop an evidence-based expert consensus to address key clinical dilemmas inherent to DAA-HJA in geriatric FNF. Sponsored by the Joint Surgery Group of the Chinese Medical Association, this expert consensus builds upon the foundational Chinese Expert Consensus on Direct Anterior Approach Hip Arthroplasty for the Surgical Treatment of Geriatric Femoral Neck Fracture (2023 Edition). A multidisciplinary expert panel was convened to standardize core issues and procedural norms for DAA-HJA in geriatric FNF. Two rounds of modified Delphi questionnaires and one consensus conference were conducted for voting, with a predefined consensus threshold of ≥ 70%. A total of 12 clinical recommendations were formulated, covering critical concerns including surgical indications, approach selection, operative timing, anesthesia management, patient positioning, prosthesis choice, fixation methods, drainage strategies, postoperative mobilization, and the application of navigation/robotic technologies. These recommendations are stratified by strength: Recommendations 4, 8, and 12 are classified as strong; Recommendations 1, 3, 5, and 9 are moderate; and Recommendations 2, 6, 7, 10, and 11 are limited. By providing systematic, evidence-based, and operationally feasible guidance for the clinical application of DAA in geriatric FNF, this consensus aims to promote standardized, systematic, and individualized diagnosis and treatment paradigms—ultimately optimizing patients’ functional prognosis.

Video Abstract

**Supplementary Information:**

The online version contains supplementary material available at 10.1186/s42836-026-00368-9.

## Introduction

Femoral neck fracture (FNF) constitutes the majority of hip fractures in the elderly, and it is a universally recognized medical challenge that transcends public health, economic culture, and impacts national development strategies. Especially amidst China’s accelerating aging population, national development transformation, and the impact of the COVID-19 pandemic on the international landscape, it is essential to re-evaluate the impact of “the last fracture of life” on families, society, and national development. Studies predict that by 2050, there will be 6.3 million elderly hip fracture patients worldwide, with over 50% occurring in Asia [1]. An analysis of 27,205 elderly hip fracture cases from 73 tertiary hospitals in 24 provinces in China found that the treatment costs and medical resource consumption for 60 ~ 69-year were significantly higher than those for patients aged over 80 years and under 60-year [2], suggesting an urgent need for optimization in the treatment of elderly hip fractures between 60 and 80 years old.

Currently, there are controversies regarding the treatment options, including surgical indications, timing, and approaches for elderly FNF. However, a large number of clinical studies and guideline consensus have recommended hip joint arthroplasty (HJA) as the primary choice [3, 4] attributing to its advantages of reducing readmission rates, disability-free survival rates, and improving quality of life scores. Given the limitations of traditional hip arthroplasty techniques, such as posterior lateral and anterior lateral approaches, which include instability and dislocation [5], gluteus medius interference, lower limb length discrepancy, and gait abnormalities, direct anterior approach (DAA) hip arthroplasty (DAA-HJA) has emerged as a potential solution in addressing the challenges of elderly FNF. According to a 2024 survey conducted by the American Association of Hip and Knee Surgeons (AAHKS), 70% of surgeons now employ the DAA in hip arthroplasty procedures [6, 7]. In Europe, data specific to the Netherlands indicates that the DAA adoption rate has risen steadily from 0.2% in 2007 to 41% in 2020 [8].

Notably, the National Institute for Health and Care Excellence (NICE 2023) [9] and the American Academy of Orthopaedic Surgeons (AAOS 2021) [10] have provided detailed elaborations and recommendations on geriatric hip fractures, covering a wide range of aspects including imaging examinations, analgesic strategies, surgical timing and approach, anesthesia selection, prosthetic fixation modalities, and postoperative patient management. These guidelines offer evidence-based support for the diagnosis and treatment of hip fractures in elderly patients. Due to the discrepancy in ethnicity, culture, and policy, there is still an absence of evidence on the Chinese population.

In recent years, robot-assisted (RA)-HJA has gained popularity in developed countries in Europe and America [11, 12], with higher accuracy in component implantation and lower incidences of postoperative complications [13]. This approach better caters to the needs of elderly patients for early weight-bearing ambulation and early recovery of family and social functions. However, due to the widespread influence of the traditional approach and the challenges associated with the learning curve of DAA [14], the popularization of DAA-HJA and RA-HJA in China is uncertain. Therefore, this expert consensus revises the “Chinese Expert Consensus on the Surgical Treatment of Femoral Neck Fracture by Direct Anterior Approach Hip Arthroplasty for Elderly Patient (2023 Edition)”, focusing on supplementing high-evidence research and high-quality literature in the field of RA-DAA-HJA. In general, twelve recommendations are proposed from five perspectives: indications, key techniques, operational procedures, perioperative management, robot and navigation technologies, aiming to further standardize and efficiently promote DAA-HJA.

## Methods

### Consensus source and working group constitution

This expert consensus was initiated by the Hip Joint Group of the Orthopedic Society of the Chinese Medical Association (JSG-COA). A total of 60 experts in the field of adult hip reconstructive surgery from 22 provinces, municipalities, and autonomous regions in China were recruited to form a guideline working group. Included experts needed to meet at least two of the following criteria based on clinical qualifications and informed consent: (1) performed DAA hip arthroplasty for ≥ 5 years; (2) completed at least 200 DAA surgeries annually; (3) cumulatively completed over 500 hip surgeries for elderly femoral neck fractures; (4) completed over 100 robotic-assisted joint surgeries.

### Source of consensus items and literature screening analysis

**Fig. 1:**
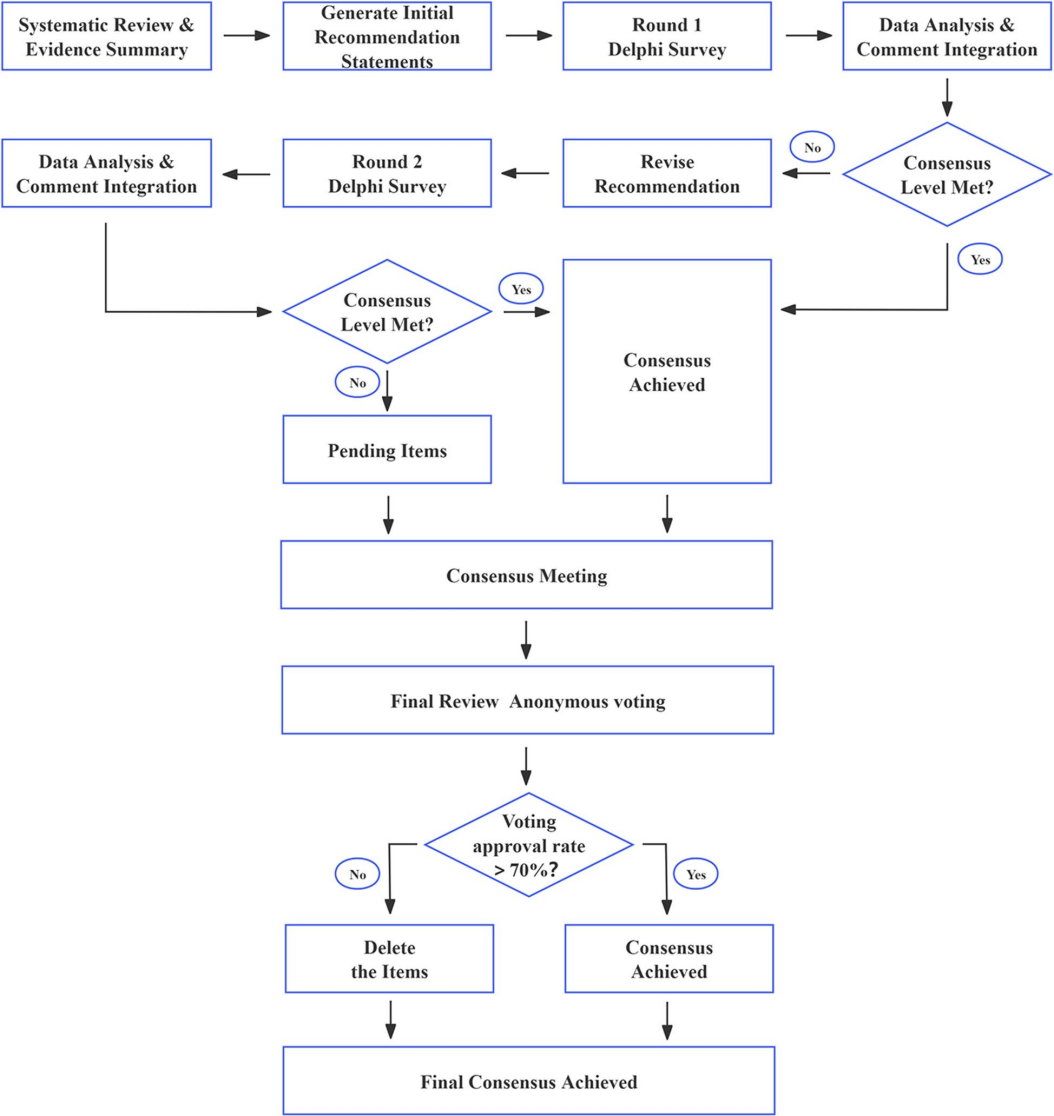
Development workflow of the expert consensus

The literature retrieval databases included PubMed, Web of Science, Embase, Cochrane Library, China National Knowledge Infrastructure (CNKI), Wanfang Data, and VIP Information. The search keywords included “elderly”, “high age”, “femoral neck fracture”, “direct anterior approach”, “hip replacement/arthroplasty”, and “robot”. The literature inclusion criteria were clinical studies related to direct anterior approach hip arthroplasty for elderly FNF, including systematic reviews/meta-analyses, randomized controlled trials (RCTs), non-randomized controlled trials, clinical trials, cohort studies, case–control studies, case reports, and cross-sectional studies. The exclusion criteria were: (1) duplicate content; (2) non-English or Chinese studies; (3) conference abstracts, short articles, reviews, etc.; (4) inability to determine study type and level of evidence. The search period was from January 2000 to August 2025. A total of 578 articles were retrieved, including 482 in English and 96 in Chinese, with 74 articles (71 in English and 3 in Chinese) ultimately cited, including 19 systematic reviews, 15 RCTs, 17 cohort studies, 22 reviews, and 1 questionnaire survey. The workflow of consensus development is illustrated in Fig. 1.

### Evidence grading and recommendation strength

Evidence grading was conducted by experts in evidence-based medicine, with recommendations based on the highest level of evidence. The evidence level was determined using the UK Cochrane Center evidence grading system [15]. The recommendation strength was divided into three levels based on the voting agreement rate among experts: 100% complete agreement (strong), 90% ~ 99% strong agreement (moderate), and 70% ~ 89% agreement (limited)***.***

### Process of consensus formation

In accordance with the “Guiding Principles for Formulating/Revising Clinical Practice Guidelines in China (2022 Edition)” published in the Chinese Medical Journal [16], a three-step utilized Delphi method was employed during the implementation process. This included 17 preliminary recommendations categorized into five major categories. Based on literature retrieval support and two rounds of questionnaires, these were further refined into 12 final recommendations. This consensus was registered and published in the Chinese Clinical Trial Registry (PREPARE-2023CN044).

## Results

### Recommendation 1. Surgical indications

Patients with intra-capsular displaced fracture (Garden type III ~ IV), poor bone quality, multiple medical comorbidities, or a high risk of failure with internal fixation (IF) surgery should consider DAA-HJA for maximum clinical benefit. (Consensus recommendation: strong; Evidence level: B).

To evaluate the indications for DAA-HJA in the treatment of FNF in elderly patients, seven recommended articles were reviewed. Bartels et al. [17] conducted a multicenter RCT study on patients aged 55 ~ 70 with low-energy displacement FNF and found that THA achieved higher functional scores, including Harris Hip Score (HHS), Oxford Hip Score (OHS), Hip Disability and Osteoarthritis Outcome Score (HOOS), and EuroQoL 5-Dimension 3-Level (EQ5D3L), starting from 4 months postoperatively compared to internal fixation (IF). Wilson et al. [18] matched 778 cases of 49-year-olds and 3470 cases of 50 ~ 59-year-olds with FNF (IF versus THA) and found that the reoperation rate for IF was significantly higher than THA within 3 years postoperatively (OR: 2.35 at 1 year, OR: 5.86 at 3 years). Among the studies using Garden classification, whether femoral head arthroplasty (FHA) [19], THA [20, 21] or dual-mobility arthroplasty [22], elderly patients with Garden III and IV FNF were identified as the primary applicable group. Therefore, a detailed preoperative assessment of the patient’s general condition, underlying diseases, bone quality, soft tissue status, cognitive function, and activity level is necessary to objectively evaluate surgical expectations, thereby choosing a more individualized surgical plan [23].

### Recommendation 2. Approach selection

All the current surgical approaches have demonstrated favorable clinical outcomes. The DAA-THA is recommended for specialized hip surgeons who possess a robust foundation in traditional hip surgery, have completed specialized DAA training, and have acquired sufficient experience to overcome the associated learning curve issue. (Consensus recommendation: moderate; Evidence level: B).

Multiple surgical approaches are available for THA, most commonly the Posterolateral Approach (PLA), Direct Lateral Approach (DLA), and DAA, each with distinct pros and cons. The PLA offers straightforward exposure and surgeon familiarity but carries a relatively higher risk of dislocation; the DLA may be associated with hip adductor interference and increased blood loss; and the DAA yields greater early patient-reported outcome improvements but tends to involve longer operative times [24]. To evaluate the efficacy of the aforementioned surgical approaches for THA in Chinese geriatric patients with FNF, six studies were included in the analysis.

A meta-analysis by Awad et al. [25] (29 clinical studies) demonstrated that compared with the PLA, the DAA had significantly shorter incision length [(11.3 ± 1.7) cm vs. (14.7 ± 2.6) cm, *P* < 0.001] and hospital stay [(2.85 ± 0.89) days vs. (3.30 ± 1.09) days, *P* < 0.001], but longer operative time [(97.7 ± 20.7) min vs. (88.3 ± 23.3) min, *P* < 0.001] and increased intraoperative blood loss [(430.6 ± 175.8) mL vs. (330.3 ± 171.2) mL, *P* = 0.03]. The DAA also showed superior Harris Hip Scores (HHS) at 3 weeks (*P* < 0.001) and 6 weeks (*P* = 0.006) postoperatively, with no significant differences vs. PA from 6 weeks to 1 year (all *P* > 0.1), indicating short-term (≤ 6 weeks) benefits of DAA. A systematic review by Yan [26] (63 RCTs) corroborated these findings, noting no interapproach safety differences. Morgan et al.’s retrospective study [27] (164 patients) reported that the direct lateral approach (DLA) group had shorter operative time (2.3 vs. 2.8 h, *P* = 0.03), longer hospital stay (5.0 vs. 4.0 days, *P* < 0.01), and less intraoperative blood loss (50 vs. 100 mL, *P* = 0.01) vs. PA. Bischofreiter et al. [28] found that five surgeons achieved consistent DLA-THA outcomes after 30 cases each, while Nairn et al.’s [14] systematic review indicated DAA-THA operative time stabilized after ~ 100 cases, reflecting a longer learning curve for DAA. A consensus statement from the 2024 World Expert Meeting in Arthroplasty recommended that, in addition to patients’ individual characteristics, surgeons should consider their own proficiency with a given technique when selecting a surgical approach for THA [24].

### Recommendation 3. Timing of surgery

For elderly patients with FNF scheduled for DAA-THA, surgical intervention is recommended to be initiated preferably within 48 h of admission after a comprehensive surgical risk assessment and strict exclusion of contraindications to optimize clinical outcomes. (Consensus recommendation: moderate; Evidence level: B).

To evaluate the optimal timing of surgery for elderly patients with FNF, two studies were included. A meta-analysis by Chen et al. [29] (27 clinical studies, 33,727 participants) demonstrated that for patients with proximal femoral fractures, surgical intervention within 48 h of injury was associated with a 28% reduction in mortality risk compared to surgery performed after 48 h (RR: 0.72; 95% CI: 0.71–0.73). Similarly, surgery conducted within 24 h resulted in a 23% lower mortality rate relative to later intervention (RR: 0.77, 95% CI: 0.65–0.93). Furthermore, early surgery was linked to a lower incidence of perioperative complications compared to delayed surgery (OR: 0.52, 95% CI: 0.35–0.76). A retrospective study by Su et al. [30] analyzed 243 hip fracture patients, among whom 96 underwent surgery within 48 h of admission and 147 after 48 h of admission. The results showed that the incidence of anemia in the early surgery group was significantly lower than that in the delayed surgery group (60.42% vs 72.79%, *P* = 0.043). Collectively, these studies indicate that early surgical intervention confers substantial benefits to elderly hip fracture patients. Therefore, for elderly patients with FNF scheduled for DAA-THA, surgical treatment is recommended to be prioritized and initiated within 48 h of hospital admission, following a comprehensive surgical risk assessment and strict exclusion of contraindications, to optimize clinical outcomes.

### Recommendation 4. Anesthesia

The choice of anesthesia for elderly patients of FNF undergoing DAA-HJA should be based on individual patient circumstances, available technical resources, and surgical platform turnover efficiency, with personalized selection. (Consensus recommendation: strong; Evidence level: A).

To evaluate anesthesia type for elderly FNF patients undergoing DAA-HJA, three studies were included. Neuman et al.’s [31] global multicenter RCT (REGAIN) (1,600 hip fracture patients: 795 spinal anesthesia [SA], 805 general anesthesia [GA]) found no significant differences in 60-day mortality, new inability to walk independently (RR: 1.03, 95% CI: 0.84–1.27, *P* = 0.83), or postoperative delirium between groups. A Cochrane systematic review [32] (31 RCTs, 3,231 patients) confirmed no differences in 30-day major outcomes (mortality, pneumonia, myocardial infarction, cerebrovascular events, delirium, deep venous thrombosis) between SA and GA. A retrospective analysis [33] reported no variances in postoperative cognitive function, 30/60-day mortality, or ambulation among GA, SA, and combined spinal-epidural anesthesia (CSEA); however, SA/CSEA significantly reduced hospital stay, 90-day mortality, pulmonary complications, severe intraoperative hypotension, and acute kidney injury risk vs. GA. These findings indicate no clinical outcome disparities between SA and GA for elderly FNF patients undergoing DAA-HJA, but hemodynamic considerations (e.g., obese patients with abdominal panniculus, heart failure, chronic obstructive pulmonary disease) must be addressed. Anesthesia selection should be personalized based on patient characteristics, technical resources, and surgical efficiency.

### Recommendation 5. Supine versus lateral position

Both supine and lateral decubitus positions for DAA-HJA can meet the needs for minimally invasive surgery and accelerated recovery. (Consensus recommendation: moderate; Evidence level: D).

Recently, some Chinese scholars have reported that DAA-HJA performed in the lateral decubitus position can achieve similar clinical outcomes to those in the supine position. To assess the surgical outcomes of DAA-HJA in both positions, five recommended articles were reviewed. Zhao et al. [34] retrospectively compared the accuracy of acetabular position between lateral decubitus DAA (46 cases) and supine DAA (43 cases), and found no statistical differences in anteversion and abduction angles. An RCT study by Xiao et al. [35] (54 supine versus 36 lateral decubitus) showed no differences in operation time, hospital stay, and blood loss between the two groups, with satisfactory postoperative functional and radiological outcomes. Liu et al. [36] retrospectively analyzed 94 DAA-THA patients (45 lateral decubitus versus 49 supine) and found that supine DAA had short-term advantages such as smaller incisions, less blood loss, shorter operation time, earlier ambulation, shorter hospital stay, and smaller errors in intraoperative acetabular position compared to lateral decubitus DAA, but there were no significant differences in outcomes at 6 months postoperatively. Xu et al.’s [37] fluoroscopic measurements found that supine DAA better replicates the patient’s physiological hip-spine adaptation and simulates the weight-bearing functional position of the pelvis, thereby achieving more precise acetabular implantation and a higher proportion of implants within the Lewinnek and Callanan safe zones. Therefore, DAA-HJA via either supine or lateral decubitus position can meet the requirements of minimally invasive surgery through the neuromuscular interval and postoperative rapid recovery [38].

### Recommendation 6. *Hemiarthroplasty versus THA*

Both DAA-HA and THA can achieve satisfactory clinical outcomes for the elderly patients of FNF, but THA offers better functional recovery and quality of life. (Consensus recommendation: limited; Evidence level: A).

To clarify the differences in outcomes between THA and HA in elderly FNF surgeries, five recommended articles were reviewed. A meta-analysis by Lewis et al. [21], 1364 patients from 17 studies showed that hemiarthroplasty (HA) had advantages in shorter operation time, lower hip dislocation rates, and overall complication rates within 4 years postoperatively. A systematic review by Tang et al. [39], based on 25 RCTs, found that HA performed better in reducing hospital stay, operation time, and blood loss, with a lower dislocation rate, while THA offered better mid-term functional outcomes and quality of life with a lower acetabular erosion rate. Ekhtiari et al. [40] conducted a meta-analysis of four RCTs and found that although HA and THA may lead to similar revision rates, functional outcomes, mortality rates, periprosthetic fractures, and dislocations within 5 years, THA provided significant benefits in health-related quality of life for FNF patients over 69 years old at the follow-up endpoint (95% CI: 0.02–0.07). Liu et al. [41] conducted a meta-analysis of nine RCTs and showed that THA provided superior hip function and quality of life with controllable risks, recommended as the preferred management option for active elderly patients over 75 years old. Conclusively, THA and HA have similar clinical outcomes in terms of functional recovery, complications, revision rates, and mortality. A consensus statement from the 2024 World Expert Meeting in Arthroplasty concluded that, with up to 5 years of follow-up, no clinical differences exist between HA and THA in the treatment of elderly patients with displaced FNF. However, THA may be preferentially considered for younger, more active patients [42].

### Recommendation 7. Bone cement versus cementless fixation

Both cemented and cementless femoral prostheses can achieve satisfactory clinical outcomes in elderly patients with FNF undergoing DAA-THA. However, cemented femoral prostheses are beneficial for reducing early postoperative complications and improving patient satisfaction in populations with severe osteoporosis, special medullary canal morphology, or other similar conditions. (Consensus recommendation: limited; Evidence level: A).

To compare the clinical efficacy differences between cemented and cementless (biological) femoral stem prostheses, a database search was conducted, and a total of six relevant studies were included. The overall conclusion is that cemented femoral prostheses achieve superior long-term outcomes in the elderly population, with reduced risks of periprosthetic fractures and revision surgery. Fernandez et al. [43] conducted a multicenter randomized controlled trial (RCT) comparing the efficacy of hip arthroplasty via the DAA in 1,225 patients aged ≥ 60 years with intracapsular FNF. The study demonstrated that patients who underwent cemented hip arthroplasty experienced significantly greater improvements in quality of life and a lower risk of periprosthetic fractures compared to those who received cementless hip arthroplasty (cemented group vs. cementless group: 0.5% vs. 2.1%, OR: 4.37, 95% CI: 1.19–24.00). Axenhus et al. [44] performed an RCT to evaluate the long-term outcomes of cemented versus cementless femoral stem total hip arthroplasty (THA) in 69 patients aged 65–79 years with displaced FNF. At 10-year follow-up, the cemented fixation group showed a significantly lower risk of reoperation compared to the cementless group (5% vs. 21%, *P* < 0.001). A meta-analysis by Imam et al. [45], which included 29 studies (9 RCTs and 20 observational studies) involving 42,046 hips, revealed that the cemented prosthesis group had fewer intraoperative and postoperative fractures (RR: 0.44, 95% CI: 0.21–0.91). However, this group was associated with longer surgical duration, increased intraoperative blood loss, and a higher incidence of heterotopic ossification. A retrospective study by Olsen et al. [46] enrolled 1,095 patients who underwent hip arthroplasty for displaced FNF, including 986 patients with cemented cylindrical femoral stems and 109 with cementless femoral stems. The results indicated that the cemented femoral stem group had a higher incidence of hypoxia and/or hypotension (28% vs. 17%, *P* = 0.003), and the use of cemented femoral stems was identified as an independent risk factor for 1-year mortality (HR: 1.9, 95% CI: 1.3–2.7).

Furthermore, caution is warranted regarding the risk of bone cement implantation syndrome (BCIS) when utilizing cemented femoral stems [47], and proactive preventive strategies should be implemented. Nevertheless, cemented femoral prostheses offer benefits in reducing early postoperative complications and enhancing patient satisfaction in select patient populations. Consistent with this, a consensus statement from the 2024 World Expert Meeting in Arthroplasty recommended the use of a cemented femoral component for women older than 70 years of age, in patients who have femoral neck fractures, in patients who have a Dorr type C femur, and in patients who have severe osteoporosis [48].

### Recommendation 8. Drainage management

The decision of whether to place a drain during DAA-HJA for elderly FNF patients should be based on individual patient factors and the specific surgical situation. (Consensus recommendation strength: strong; Evidence level: A).

With advancements in accelerated recovery, particularly in blood management and minimally invasive techniques, perioperative blood loss in HJA has significantly decreased. To address the question of whether drains should be used during DAA-HJA for elderly FNF patients, three recommended articles were reviewed. An RCT study by Suarez et al. [49] concluded that using a drain during DAA-HJA did not have a significant impact on perioperative blood loss, transfusion rates, wound complications, or hospital stay. Xu et al. [50] used a regression analysis model to analyze 6667 Chinese patients undergoing THA and found that drain placement was an independent influencing factor for increased transfusion rates and hospital stay (RR: 1.872, 95% CI: 1.588–2.207, *P* < 0.001), increasing transfusion rates by an average of 6.96% and hospital stay by 0.93 days on average. Kleinert et al. [51] also confirmed through a prospective study that continuous negative pressure drainage had no statistical relationship with perioperative hemoglobin loss and transfusion rates in 120 patients undergoing DAA-THA with cementless implant, and that the absence of drains did not significantly affect functional recovery at three months postoperatively due to thigh swelling and early pain. Therefore, for elderly FNF patients undergoing DAA-HJA, the decision on whether to place a drain should be made based on coagulation function, bleeding risk, soft tissue release extent, and the surgeon’s experience.

### Recommendation 9. Postoperative mobilization

Low-level mobilization precautions are recommended postoperatively for elderly FNF patients undergoing DAA hip arthroplasty, except for those at high risk of dislocation. (Consensus recommendation: moderate; Evidence level: B).

To evaluate the postoperative mobilization precautions for preventing dislocation and their duration and methods in elderly FNF patients undergoing DAA-HJA, seven recommended articles were reviewed. Approximately 60% of North American doctors and 71% of Nordic doctors do not support mobilization precautions for patients undergoing DAA-HJA. A questionnaire survey by Carli et al. [52] based on the American Association of Hip and Knee Surgeons showed that the main factors influencing postoperative mobilization precautions after THA were surgical approach, surgeon experience, and femoral head diameter. Approximately 63.2% of patients received mobilization precautions after posterior lateral approach THA, while the use of mobilization precautions and related equipment after DAA was significantly reduced (16.8%). Additionally, Berstock et al. [53] found poor patient compliance (only 22.6% of patients fully adhered to postoperative mobilization precautions), and the impact on patients’ sleep and daily activities increased the difficulty of managing these restrictions. Conversely, patients with no postoperative mobilization restrictions had better functional outcomes [54, 55], and eliminating mobilization precautions did not increase the dislocation rate [56, 57]. However, for patients at high risk of dislocation (such as those with sequelae of cerebrovascular disease, neuromuscular diseases, cognitive disorders, or loss of hip-spine adaptation), strict mobilization precautions are beneficial [58].

### Recommendation 10. Navigation technique

Conventional navigation remains a well-established, universally applicable technique in THA with enduring clinical value in modern surgical systems. It synergizes with advanced equipment to support diagnosis and treatment in high-level medical centers while adapting to routine application in primary institutions, offering practical operational guidance for primary care physicians. (Consensus recommendation: moderate; Evidence level: B).

Conventional computer-assisted navigation technology remains the foundation for achieving precise prosthesis placement in THA. To clarify the value of conventional navigation in THA, a total of five studies were included. A meta-analysis of randomized controlled trials (RCTs) by Xu et al. [59], which incorporated 13 studies involving 1,071 hips, demonstrated statistically significant differences between the navigation group and the conventional group in terms of the proportion of acetabular cups placed outside the safe zone (RR: 0.13, 95% CI: 0.08–0.22, *P* < 0.00001), surgical time (MD = 19.87 min, 95% CI: 14.04–24.35, *P* < 0.00001), and leg length discrepancy (MD = − 4.61 mm, 95% CI: − 7.74– − 1.48, *P* = 0.004). However, no significant differences were observed in cup inclination, anteversion, postoperative dislocation rate, or deep vein thrombosis (DVT) incidence between the two groups. Additionally, a meta-analysis by Liu et al. [60] involving 7 studies and 485 patients found that in THA, image-free navigation was associated with a significantly reduced relative risk of acetabular cup malposition outside the safe zone compared to conventional methods (RR: 0.31, 95% CI:0.17–0.55, *P* < 0.0001). Similar conclusions were drawn from two other meta-analyses by Migliorini [61], Miura [62], et al.A retrospective study by Agarwal et al. [63] including 6,912 THA patients revealed that the all-cause revision rate in the navigation group was significantly lower than that in the non-navigation group (HR: 0.64, 95% CI: 0.48–0.86, *P* = 0.003). Collectively, these studies indicate that conventional navigation technology offers certain advantages over non-navigation approaches.

### Recommendation 11. Robotic surgery

Robot-assisted (RA)-DAA-HJA might decrease intraoperative fluoroscopic radiation exposure for surgeons, patients, and operating room staff. (Consensus recommendation: limited; Evidence level: B).

X-ray fluoroscopy is frequently used to confirm the prosthesis implantation, limb length, offset, etc. in traditional HJA; therefore, radiation exposure for the surgeon, patient, and operating room staff is a significant concern. To evaluate the duration and dose of radiation exposure during HJA, three recommended articles were reviewed. Curtin et al. [64] conducted a multicenter retrospective analysis of 157 patients undergoing unilateral DAA-THA and found that the average radiation dose for patients was (2.97 ± 1.63) mGy, almost identical to the value for screening mammography (3 mGy) and one-fourth of the standard chest CT radiation dose (13 mGy). Another retrospective analysis by Neitzke et al. [65] included 6,541 patients (4,333 traditional DAA-THA, 1,158 RA-THA, and 1,050 CT-navigated THA) undergoing unilateral DAA-THA. They found that the robot technique reduced the average fluoroscopy time and radiation dose by 78.9% and 84.6%, respectively, compared to traditional and CT-navigated THA. Sequeria et al. [66] found that in six cadavers undergoing DAA-THA guided by fluoroscopy and RA-DAA-THA, the former required an average of 21 ± 8.9 fluoroscopic views during surgery, with an average radiation dose of 300 ~ 1033mrem, while the latter had an average preoperative CT radiation dose of 289 mrem. These results significantly reduced concerns about radiation exposure for surgeons and operating room staff without increasing patient radiation exposure.

### Recommendation 12. Robotic surgery

RA-DAA-HJA can enhance the accuracy, precision of component placement, and surgical efficiency. (Consensus recommendation: strong; Evidence level: A).

To assess the accuracy of prosthesis placement in RA-DAA-HJA, six recommended studies were reviewed. Xu et al. [67] demonstrated that the proportion of acetabular components within the Callanan and Lewinnek safe zones was 56% and 72% for traditional DAA, respectively, compared with 72% and 100% for RA-DAA (*P* < 0.05). Kunze et al. [68] conducted a matched-pair analysis comparing robotic-assisted DAA and posterior approach (PA) in 134 total hip arthroplasty (THA) patients; while no statistical difference in acetabular anteversion was observed between the two groups, the DAA cohort exhibited greater precision (*P* = 0.001), with the PA group showing higher radiological deviation (*P* = 0.016). Zhang et al. [69] performed a multicenter randomized controlled trial (RCT) comparing RA-THA via anterior, lateral, and posterior approaches, reporting minimal discrepancies in acetabular rotation center offset and bilateral limb length for the anterior approach (*P* < 0.001). Emara et al.’s meta-analysis [70] revealed a significantly smaller angle difference between the femoral component and the native femur in the RA-THA group compared with traditional THA (*P* = 0.001). Caba et al. [71] conducted paired RA and fluoroscopy-guided DAA-THA on six cadavers (12 hips), finding that the robotic group achieved acetabular reaming in a single attempt per preoperative planning—simplifying the procedure compared with the traditional technique, which typically requires graded reaming (mean 2.67 ± 0.5 attempts). Notably, the definition of “safe zone” should not be confined to the traditional Callanan and Lewinnek criteria; practitioners performing DAA-THA should shift their focus to the hip-spine motion and pelvic functional alignment [72].

Despite these advantages, a consensus statement from the 2024 World Expert Meeting in Arthroplasty concluded that robotic-assisted total hip arthroplasty (RA-THA) does not offer clear clinical outcome advantages over conventional total hip arthroplasty (cTHA). Further high-quality studies are warranted to confirm whether the improved radiographic precision achieved with robotic surgery translates to superior clinical outcomes and long-term prosthesis survivorship.

## Discussion

This consensus was endorsed by the Joint Surgery Group of the Chinese Medical Association (JSG-COA) and jointly organized by the Joint Surgery Branch of Chongqing Medical Association. It focuses on core clinical issues and standardized procedural protocols for the surgical management of geriatric femoral neck fractures (FNF), intending to formulate targeted, evidence-based clinical recommendations. In terms of content, it supplements the 2023 guidelines issued by the National Institute for Health and Care Excellence (NICE) and the 2021 clinical practice guidelines from the American Academy of Orthopaedic Surgeons (AAOS). A comparative analysis of the three documents is summarized in Table 1. In terms of content, this consensus supplements the 2023 clinical guideline on hip fracture management from the National Institute for Health and Care Excellence (NICE), the 2021 clinical practice guideline for older adults with hip fractures from the American Academy of Orthopaedic Surgeons (AAOS), and the consensus statements from the 2024 World Expert Meeting in Arthroplasty. Specifically, a comparative analysis of core topics across the JSG-COA consensus, NICE guideline, and AAOS guideline is summarized in Table 1.

**Table 1:** Comparison of expert consensus and clinical guidelines on DAA hip arthroplasty for surgical management of geriatric FNF: JSG-COA vs. NICE vs. AAOS

Consensus Items	JSG-COA	NICE	AAOS
Surgical Indications	Recommended	Recommended	Recommended
Approach Selection	Recommended	Recommended	Recommended
Timing of Surgery	Recommended	Recommended	Recommended
Anesthesia	Recommended	Recommended	Recommended
Postoperative Mobilization	Recommended	Recommended	Recommended
Prosthesis Selection	Recommended	Recommended	Recommended
Stem Fixation	Recommended	Recommended	Recommended
Imaging Options	Not Mentioned	Recommended	Recommended
Analgesia	Not Mentioned	Recommended	Recommended
Fracture Complication	Not Mentioned	Recommended	Not Mentioned
Preoperative Planning	Not Mentioned	Recommended	Not Mentioned
MDT	Not Mentioned	Recommended	Not Mentioned
Patient and Carer Information	Not Mentioned	Recommended	Not Mentioned
Preoperative Traction	Not Mentioned	Not Mentioned	Recommended
VTE Prophylaxis	Not Mentioned	Not Mentioned	Recommended
Blood Transfusion	Not Mentioned	Not Mentioned	Recommended
Drainage Management*	**Recommended**	**Not Mentioned**	**Not Mentioned**
Navigation Technique*	**Recommended**	**Not Mentioned**	**Not Mentioned**
Robotic Surgery*	**Recommended**	**Not Mentioned**	**Not Mentioned**

^*^The bolded content represents newly developed items that have not been recommended in the NICE or AAOS guidelines

A key strength of this expert consensus lies in its specific reference to robotic surgery in DAA-THA for geriatric femoral neck fractures. However, it is important to acknowledge that despite the advantages and supporting data outlined in Recommendations 11 and 12, evidence demonstrating that robotic technology confers improved functional outcomes following THA remains very limited. This aligns with a consensus statement from the 2024 World Expert Meeting in Arthroplasty, which concluded that RA-THA does not offer clear clinical outcome advantages over conventional THA. Consequently, further high-quality studies, preferably large-scale randomized controlled trials with long-term follow-up, are warranted to confirm whether the well-documented improvements in radiographic precision associated with robotic surgery translate to superior clinical outcomes and enhanced long-term prosthesis survivorship [73].

To ensure the effective implementation of this consensus, careful execution and ongoing auditing are recommended, which were supported by the following key measures:

1. Facilitating a Smooth Learning Curve Transition. Surgeons and medical institutions should acknowledge that the DAA entails a well-documented learning curve. Proficiency plateau, characterized by stable operative times, reduced fluoroscopy utilization, and diminished complication rates, is typically achieved after 50–100 procedures. During the initial learning phase, the risk of complications (e.g., femoral stem fracture, greater trochanteric fracture) may be elevated. To navigate this phase safely, simulation training, cadaveric workshops, surgical observation, and early clinical cases performed under the supervision of experienced surgeons are recommended.
2. Implementing a Perioperative Safety Checklist. Adoption of a standardized perioperative safety checklist is advised to preclude oversight of critical steps, thereby enhancing interdisciplinary team collaboration and patient safety. This checklist should encompass all stages from preoperative assessment to discharge planning, with integrated risk prevention and control strategies for bone cement implantation syndrome (BCIS).
3. Promoting Timely and Accurate Data Reporting. The expert panel strongly advocates for all relevant medical institutions and surgeons to routinely submit comprehensive, accurate data—including baseline patient characteristics, surgical details, and follow-up outcomes—for every hip arthroplasty procedure to the National Joint Registry. Such data submission serves as the foundation for large-scale population health outcomes monitoring, prosthetic performance evaluation, and long-term comparative effectiveness research on surgical techniques, which are pivotal to advancing the national standard of diagnosis and treatment for hip fractures.

## Conclusion

This consensus articulates the rationale for performing DAA hip arthroplasty in geriatric patients with FNF. It synthesizes key evidence on surgical indications, core technical principles, perioperative management strategies, and the clinical application of robotic technologies for this patient population. These recommendations are formulated to serve as a clinical practice reference and to facilitate the standardization of surgical procedures. It is important to emphasize that this consensus constitutes an academic guideline rather than a legal mandate. Given the unique characteristics of robotic technology, including inherent ethical considerations and associated learning curve, hip surgeons are advised to conduct a comprehensive assessment of institutional resources and individual patient factors when implementing these recommendations in clinical practice.

## Data Availability

No datasets were generated or analysed during the current study.

## Abbreviations

FNF: Femoral neck fracture
DAA: Direct anterior approach
HJA: Hip joint arthroplasty
THA: Total hip arthroplasty
HA: Hemiarthroplasty
RA: Robotic technology
